# Pre- and Post-Operative Rehabilitation Interventions in Patients at Risk of Poor Outcomes Following Knee or Hip Arthroplasty: Protocol for Two Systematic Reviews

**DOI:** 10.1177/27536351231170956

**Published:** 2023-05-10

**Authors:** Motahareh Karimijashni, Samantha Yoo, Keely Barnes, Stéphane Poitras

**Affiliations:** 1 School of Rehabilitation Sciences, Faculty of Health Sciences, University of Ottawa, Ottawa, ON, Canada; 2 Ottawa Hospital Research Institute, Ottawa, ON, Canada; 3 School of Epidemiology and Public Health, Faculty of Medicine, University of Ottawa, Ottawa, ON, Canada; 4 Bruyère Research Institute, Ottawa, ON, Canada

**Keywords:** Total joint arthroplasty, osteoarthritis, rehabilitation, poor outcome

## Abstract

**Objective:**

Total knee (TKA) and hip arthroplasty (THA) are successful procedures in treating end-stage osteoarthritis when nonoperative treatments fail. However, a growing body of literature has been reporting suboptimal outcomes following TKA and THA. While pre- and post-operative rehabilitation is imperative to recovery, little is known about their effectiveness for patients at risk of poor outcomes. In the 2 systematic reviews with identical methodology, we aim to evaluate the effectiveness of (a) pre-operative and (b) post-operative rehabilitation interventions for patients at risk of poor outcomes following TKA and THA.

**Methods:**

The 2 systematic reviews will follow the principles and recommendations outlined in the Cochrane Handbook. Only randomized controlled trials (RCTs) and pilot RCTs will be searched in 6 databases: CINAHL, MEDLINE, Embase, Web of Science, Pedro, and OTseeker. Eligible studies including patients at risk of poor outcomes and evaluating rehabilitation interventions following and preceding arthroplasty will be considered for inclusion. Primary outcomes will include performance-based tests and functional patient-reported outcome measures, and secondary outcomes will include health-related quality of life and pain. The quality of eligible RCTs will be evaluated using the Cochrane’s risk of bias tool, and the strength of evidence will be assessed using the Grades of Recommendation, Assessment, Development, and Evaluation (GRADE).

**Discussion:**

These reviews will synthesize the evidence regarding the effectiveness of pre-and post-operative rehabilitation interventions for patients at risk of poor outcomes, which in turn may inform practitioners and patients in planning and implementing the most optimal rehabilitation programs to achieve the best outcomes after arthroplasty.

**Systematic Review Registration:**

PROSPERO CRD42022355574

## Introduction

Total knee arthroplasty (TKA) and total hip arthroplasty (THA) are recognized as the most common and cost-effective elective surgical procedures for symptomatic end-stage hip and knee osteoarthritis (OA) to relieve pain and restore functional status (1). There has been an increasing demand for these surgeries over the past decade^1,2^ due to several factors including obesity, population aging, increases in sports-related injuries, changing expectations about the quality of active life, and expanding surgical indications.^3-5^ In recent years, THA and TKA cases increased 5% annually on average for both joints in Canada.^1^ Although THA and TKA are generally successful interventions, leading to improvement in pain levels and functional status, it is well documented that up to one-third of patients undergoing arthroplasty report challenges in the recovery in terms of persistent pain, restricted range of motion (ROM), functional disability, poor quality of life, and dissatisfaction.^6-8^ The patients at risk of poor outcomes are less likely to perform the activities they would like to do after surgery and more likely to be high consumers of healthcare services.

Various concepts for poor outcomes are used, and there is no consensus on its definition.^9^ A review by Te Molder et al^9^ reported that 8 different domains were used in the literature to define poor outcomes, including pain, joint function, physical functioning, quality of life, patient satisfaction, anxiety, depression, and global patient assessment. In addition, several demographic and clinical factors have been shown to be risk factors for poor outcomes such as older age,^10-13^ female gender,^10,14-16^ frailty,^17,18^ obesity,^19,20^ presence of comorbidity,^21-25^ pre-operative muscle strength,^26,27^ pre-operative ROM,^28^ pain,^29,30^ pre-operative function,^31-34^ concomitant musculoskeletal joint complaints beyond the operated joint,^29,35^ and psychological problems.^36-40^

Rehabilitation is an essential part of the provision of THA and TKA and the quality of rehabilitation strategies has been suggested to be a key determinant of post-operative outcomes.^41-43^ These strategies could be delivered to patients pre-operatively (also known as prehabilitation) and post-operatively with a goal of supporting surgical preparation and recovery. Prehabilitation and rehabilitation strategies are designed to help patients regain and maintain physical, sensory, intellectual, psychological, and social function,^6,44,45^ ensure shorter hospital stays, fewer complications, and reduced utilization of follow-up services.^46^ Rehabilitation could involve contributions from many healthcare disciplines, including physiotherapy, occupational therapy, psychology, prosthetic/orthotic, and is offered in a variety of settings (inpatient, outpatient, home supervised (including telerehabilitation and telephone interview) and unsupervised).^47-50^ Rehabilitation and prehabilitation interventions for TKA and THA can include pre- and post-operative exercises, education, non-exercise interventions (eg, cryotherapy, continuous passive motion, electrical stimulation, assistive device), gait and balance training, and psychological interventions.^6,51-53^

Despite the expanding body of knowledge on numerous post-operative interventions, the most effective way of providing rehabilitation remains unclear following TKA and THA.^41,42^ Most studies have targeted the average patients and have not focused on rehabilitation programs for patients at risk of poor outcomes.^2,54^ Because of this, some patients may not receive the most appropriate form of rehabilitation for their circumstances, resulting in suboptimal outcomes and wide variations in clinical practice.^36,41,55^ Several recent systematic reviews have evaluated the effectiveness of different rehabilitation strategies before and after TKA and THA.^43,56-58^ However, little is known on the effectiveness of rehabilitation programs for patients at risk of poor outcomes.

### Objectives

These systematic reviews aim to provide evidence regarding the effect of pre- and post-operative rehabilitation interventions on patients at risk of poor outcomes undergoing TKA and THA. Specific objective is to determine the effectiveness of pre- and post-operative rehabilitation interventions on function, quality of life, and pain for patients at risk of poor outcomes following TKA and THA.

## Methods

This protocol followed the Cochrane Handbook for Systematic Reviews^59^ and was reported based on the Preferred Reporting Items for Systematic Review and Meta-analysis Protocols (PRISMA-P) checklist guidelines (Supplemental Appendix A).^60^ The protocol for these systematic reviews was registered in PROSPERO (registration number CRD42022355574).

### Eligibility criteria

See Table 1 for the inclusion and exclusion criteria.

**Table 1.** Inclusion and exclusion criteria.

PICO framework	Inclusion	Exclusion
Population	• Adults aged 18 or older	• Unicompartmental, hip resurfacing, hemiarthroplasty
	• Primary elective knee and hip arthroplasty due to OA.	• Arthroplasty for:
	• Patients at risk of poor outcomes	○ *Rheumatoid arthritis*
	○ *Based on ICF domains (impairment, activity limitation, participation restriction outcome measures, environmental and personal factors)*	○ *Dysplasia*
		○ *Revision*
		○ *Trauma*
		○ *Other situations other than OA*
Intervention	• All types of pre- and post-operative rehabilitation interventions (non-invasive, non-medication)	• Invasive interventions
	• Without restrictions on duration	• Interventions involving pharmaceutical products
	• Any intervention setting	
	• By any healthcare providers, research staff, or supervisors	
Comparator	• No treatment	
	• Usual care	
	• Another rehabilitation intervention	
Outcome	• Primary outcome:	• Do not report any of the outcomes of interest
	○ *Performance-based measures*	
	○ *PROMs of function*	
	• Secondary outcomes:	
	○ *Health-related quality of life measures*	
	○ *Pain*	
Study design	• RCTs, Pilot RCTs	• Abstracts
		• Book chapters
		• Thesis dissertations
		• Observational studies
		• Quasi-RCTs
		• Nonrandomized clinical trials
		• Reviews
Others	• No restrictions in the date of publication	• Gray literature (eg, government reports, clinical practice guidelines)

#### Population

The current reviews will consider studies that include adults (aged 18 years and older) at risk of poor outcomes, with at least 85%^61^ undergoing primary elective TKA or THA due to OA because this surgery is predominantly considered to treat moderate to severe OA.^1,62^ Studies that include patients who underwent arthroplasty for reasons other than OA (eg, rheumatoid arthritis, dysplasia, trauma, revision), unicompartmental arthroplasty, hemiarthroplasty, hip resurfacing, and bilateral arthroplasty will be excluded. For pre-operative rehabilitation, patients who are scheduled to undergo TKA or THA will be included. Patients at risk of poor outcomes are defined according to the International Classification of Functioning, Disability, and Health (ICF) domains (Supplemental Appendix B). The ICF is a comprehensive framework of health states developed and published by the WHO in 2001. It is based on an integrative model of functioning, disability, and health and provides a standard language for describing an individual’s health state.^63-65^ This framework can contribute to broadening the understanding of different ways in which chronic conditions can affect patients’ functioning.^64^ The ICF model has 2 parts: (1) functioning/disability, which includes impairment, activity limitation, and participation restriction, and (2) contextual factors, including environmental, and personal factors.^63,64^ Patients at risk of poor outcomes are defined as including any of the following domains:

(1) Impairment: pre- and post-operative pain measure, joint mobility measures, or muscle strength measures
(2) Activity limitation: pre- and post-operative performance-based tests, disease- or site-specific patient reported outcome measures (PROMs)
(3) Participation restriction: pre- and post-operative generic PROMs
(4) Environmental factors: family, friends, caregiver support, or physical environment
(5) Personal factors: age, sex, socioeconomic situations, frailty, level of dependency on walking aids, presence of comorbidity, psychological factors or concomitant musculoskeletal joint complaints beyond the index joint

The above factors were derived from previous systematic reviews,^18,21,22,24,29,37,66-74^ clinical practice guideline,^47^ and Delphi study.^42^

Since there is consensus on the definition of poor response to arthroplasty,^9^ we will include the articles whether they defined the poor outcomes based on absolute or relative change. Therefore, there will be no restriction for the cut-off to define the patients at risk of poor outcomes.

Since there is no consensus on the definition of poor response to arthroplasty,^9^ the articles will be included whether they defined the poor outcomes based on absolute or relative change. Therefore, there will be no restriction for the cut-off to define the patients at risk of poor outcomes. The study will be eligible if they included only patients at risk of poor outcomes. Articles with mixed samples will be excluded unless they did subgroup analyses for each factor.

#### Intervention

The following interventions will be included:

(1) All pre- and post-operative rehabilitation interventions that are non-invasive and non-pharmacological (eg, exercise, electrical stimulation, cryotherapy, continuous passive motion, supporting devices, education, psychological intervention, and weight management/nutritional intervention).
(2) Interventions beginning in any period before surgery or after (eg, acute hospital, prior to discharge from hospital, post-acute)
(3) Provided by any healthcare providers.
(4) Any intervention setting (eg, land, water, inpatient, outpatient, community-, home, virtual) and supervised or unsupervised interventions.

Studies including multi-component interventions (eg, exercise and education) will also be included.

#### Comparators

Studies will be included if they investigated (1) a rehabilitation intervention compared with no intervention, (2) a rehabilitation intervention compared with usual care, (3) 2 different types of rehabilitation interventions compared head-to-head. Studies comparing more than 2 interventions will also be included.

#### Outcomes

Primary outcomes will include performance-based measures assessed with any tool (eg, 6-minute Walk Test (6 MWT), Timed Up and Go (TUG), Stair Climbing Test (SCT)), function PROMs assessed with any tool (eg, Western Ontario and McMaster Universities Arthritis Index (WOMAC), Oxford Hip (OHS), and Knee Scores (OKS)). Secondary outcomes will include health-related quality of life measures assessed with any tool (eg, 12- and 36-Item Short-Form Health Surveys (SF-36, SF-12), EuroQol-5 Dimension (EQ-5D)), and pain assessed with any tools (eg, Likert scale).^75^ There will be no restrictions regarding the time the outcomes were measured pre- and post-operatively.

#### Study types and others

These systematic reviews will only include randomized control trials (RCTs) and pilot RCTs. We will include the literature written in English, French, Korean, and Persian languages. There will be no restriction in the time of publication. Only the articles available in full text will be included.

### Data sources and search strategy

The published studies will be identified by searching in 6 electronic databases: CINAHL, MEDLINE, Embase, Web of Science, Pedro, and Otseeker. The search strategy was developed by the first author with the assistance of an experienced medical Librarian using keywords and Medical Subject Heading (MeSH) related to TKA, THA, total joint arthroplasty, rehabilitation, and RCT. The search strategy was based on 2 Cochrane reviews,^76,77^ and pre-designed search filter for RCTs were used.^59,77,78^ The full search strategy for MEDLINE can be found in Supplemental Appendix C. The search strategy was adapted for other databases with the help of the medical librarian. Additional manual screening of the reference lists of included articles will be performed to identify other potentially eligible studies.

### Study records

#### Data management and selection process

All the search results will be imported into the Covidence web-based platform for systematic review^79^ and Zotero reference management software,^80^ and duplicate studies will be removed. A 2-step process will be conducted for study screening and selection according to the inclusion and exclusion criteria. Initially, 2 independent reviewers will conduct the title and abstract screening for potentially eligible studies. Studies meeting the inclusion criteria or requiring full texts to determine eligibility will be passed to the next process. Two independent reviewers will screen the full texts to confirm eligibility. Any discrepancy between reviewers will be resolved by discussion or judged by another reviewer if no consensus is reached. The reviewers will document the selection process in Covidence with rationale for study exclusion.

#### Data extraction

Data will be extracted by 2 independent reviewers using a predetermined data extraction form. Three studies will be piloted to check the adequacy of extraction form and any necessary revisions will be made. Any disagreement will be resolved by discussion or by consulting another reviewer. The following information will be extracted: (1) general information such as title, first author, country of study, conflict of interest, funding, year of publication; (2) study details such as aim, design, inclusion and exclusion criteria, sample size; (3) study population such as demographic characteristics and criteria for inclusion of patients at risk of poor outcomes; (4) intervention characteristics such as type, setting and timing (starting point, duration), intensity/frequency of intervention, follow-up time points, and adherence; (5) outcome information such as primary and secondary outcomes, method of assessing outcomes, blinding of outcome assessment, adverse events; (6) surgical procedure (TKA, THA); THA and TKA results will be extracted and reported separately if available in the article; (7) summary of results. Whenever necessary, the study authors will be contacted for additional information. Supplemental Appendix D provides the items of the data extraction form.

#### Risk of bias assessment

Two independent reviewers will independently appraise the risk of bias using the Cochrane’s Risk of Bias Tool for Randomized Trials.^81^ The risk of bias assessment will include 7 domains: sequence generation; allocation concealment; blinding of participants and personnel; blinding of outcome assessment; incomplete outcome data; selective outcome reporting, and other biases. Potential bias in each item will be ranked high, low, or unclear risk based on the criteria provided in the tool. We will contact the authors of included articles for missing information or clarification if necessary. The rationale for these judgments, together with supporting information from the study, will be documented. Any disagreements between reviewers will be resolved by discussion or by consulting another reviewer. The risk of bias will be presented in a summary table.

#### Data synthesis

Included studies will be descriptively analyzed in a narrative review. Characteristics of included studies, such as patient demographics, patient risk factors, intervention type, outcome measurements, and follow-up period will be reported in a summary table. Primary and secondary outcomes will be synthesized, and comparisons will be made across rehabilitation interventions and comparators. Included studies for pre-and post-operative rehabilitation interventions will be divided by type of surgery (THA and TKA) since differences in outcomes have been shown.^62,82^

For post-operative rehabilitation interventions, each category will be divided based on 3 factors: (1) time: interventions that started within the first 12 weeks after TKA and THA and interventions that started after the 12-week period (the 12-week cut-off will be used since the greatest magnitude of functional improvement occurs within the first 12 weeks following surgery,^83,84^ (2) type of interventions (eg, exercise, electrotherapy, education, psychology), and (3) poor outcomes risk factors.

For pre-operative rehabilitation interventions, each category will be divided based on 2 factors: (1) type of interventions and (2) poor outcomes risk factors.

A meta-analysis will be undertaken for both reviews using a random-effects model if sufficiently homogeneous clinical trials are identified for comparison. Heterogeneity of the included studies will be measured using both Q and the *I*^2^ statistic.^85^ The level of heterogeneity will be determined based on *I*^2^ as follows: 0% to 40% unimportant heterogeneity, 30% to 60% moderate, 50% to 90% substantial, and 75% to 100% considerable heterogeneity.^60^ The results will be displayed in a forest plot. For primary or secondary outcomes measured on a continuous scale, standard deviations (SD) and 95% confidence intervals will be reported on. For dichotomous outcomes, risk ratios (RR) with 95% CI will be reported. If heterogeneity is high, we will conduct subgroup analyses stratified by criteria such as THA and TKA, and risk of bias score. Publication bias will be investigated and presented using a funnel plot and Egger’s test if more than 10 trials are pooled.^59,86^ All statistical analyses will be performed on RevMan Web^87^ by 2 independent reviewers. Sensitivity analyses will be conducted to assess the effect of studies with a high risk of bias.^88^

#### Confidence in cumulative evidence

The Grading of Recommendations Assessment, Development and Evaluation (GRADE) framework will be used to appraise the evidence, with regards to cumulative strength and quality by 2 independent reviewers.^59,89^ They will prepare “summary of findings” tables, including a grade of the overall quality of the body of evidence for each outcome. The quality of the body of evidence will be evaluated according to 5 GRADE criteria: risk of bias, inconsistency, indirectness, imprecision, and publication bias.^59^ Quality will be ranked as high, moderate, low, or very low, which will follow the definitions set out by the GRADE Working Group.^90^ Any discrepancy will be resolved by consensus or in consultation with a third review author.

## Discussion

To our knowledge, these are the first systematic reviews that aim to evaluate the effectiveness of rehabilitation interventions for patients at risk of poor outcomes. Given that poor outcome could be attributed to inappropriate rehabilitation interventions pre-and post-arthroplasty,^46,91^ we expect that the findings of these reviews will provide insights and guidance to practitioners. Ultimately, we seek to contribute to the knowledge of effective rehabilitation interventions for patients at risk of poor outcomes following TKA and THA. Furthermore, given that few studies focus on interventions for patients at risk of poor outcomes, these reviews can identify the gaps in the literature that could be addressed in future research.

The strength of these reviews is a focus on the subgroup of patients at risk of poor recovery. In addition, we are adopting a rigorous methodology, including eligibility criteria, a comprehensive systematic search strategy, study selection, data extraction, and quality appraisal of studies by 2 independent reviewers. These reviews are not without limitations. Firstly, the included studies will be limited to English, French, Korean, and Persian languages. Secondly, isolating the effect of rehabilitation interventions for elective arthroplasty results in the exclusion of studies on non-elective arthroplasty, such as fractures. Third, including only RCTs will result in less generalizability. Finally, although the comprehensive ICF framework will be used in these reviews allowing to include all potential patients at risk of poor outcomes, no standardized definition of poor outcomes exists as various concepts for poor outcomes after arthroplasty are used.

## Supplemental Material

sj-docx-1-rpo-10.1177_27536351231170956 – Supplemental material for Pre- and Post-Operative Rehabilitation Interventions in Patients at Risk of Poor Outcomes Following Knee or Hip Arthroplasty: Protocol for Two Systematic Reviews Click here for additional data file. Supplemental material, sj-docx-1-rpo-10.1177_27536351231170956 for Pre- and Post-Operative Rehabilitation Interventions in Patients at Risk of Poor Outcomes Following Knee or Hip Arthroplasty: Protocol for Two Systematic Reviews by Motahareh Karimijashni, Samantha Yoo, Keely Barnes and Stéphane Poitras in Advances in Rehabilitation Science and Practice
