# Supplementary material for: Pre- and Post-Operative Rehabilitation Interventions in Patients at Risk of Poor Outcomes Following Knee or Hip Arthroplasty: Protocol for Two Systematic Reviews
Source: Adv Rehabil Sci Pract. 2023 May 10;12:27536351231170956. doi: 10.1177/27536351231170956 (PMC10176557; doi:10.1177/27536351231170956)
Supplement: sj-docx-1-rpo-10.1177_27536351231170956 – Supplemental material for Pre- and Post-Operative Rehabilitation Interventions in Patients at Risk of Poor Outcomes Following Knee or Hip Arthroplasty: Protocol for Two Systematic Reviews [file sj-docx-1-rpo-10.1177_27536351231170956.docx]

**Appendices**

**Appendix A**. PRISMA-P 2015 Checklist

| **Checklist item** | **Information reported** | | **Line number(s)** |
| --- | --- | --- | --- |
|  | **Yes** | **No** |  |
| **ADMINISTRATIVE INFORMATION** | | | |
| **Title** | | | |
| Identify the report as a protocol of a systematic review |  |  | 4 |
| If the protocol is for an update of a previous systematic review, identify as such |  |  | - |
| If registered, provide the name of the registry (e.g., PROSPERO) and registration number in the Abstract |  |  | 57 |
| **Authors** | | | |
| Provide name, institutional affiliation, and e-mail address of all protocol authors; provide physical mailing address of corresponding author |  |  | 7-20 |
| Describe contributions of protocol authors and identify the guarantor of the review |  |  | 294-297 |
| If the protocol represents an amendment of a previously completed or published protocol, identify as such and list changes; otherwise, state plan for documenting important protocol amendments |  |  | - |
| **Support** | | | |
| Indicate sources of financial or other support for the review |  |  | 320-321 |
| Provide name for the review funder and/or sponsor |  |  | - |
| Describe roles of funder(s), sponsor(s), and/or institution(s), if any, in developing the protocol |  |  | - |
| **INTRODUCTION** | | | |
| Describe the rationale for the review in the context of what is already known |  |  | 59-102 |
| Provide an explicit statement of the question(s) the review will address with reference to participants, interventions, comparators, and outcomes (PICO) |  |  | 104-108 |
| **METHODS** | | | |
| Specify the study characteristics (e.g., PICO, study design, setting, time frame) and report characteristics (e.g., years considered, language, publication status) to be used as criteria for eligibility for the review |  |  | 116-186 |
| Describe all intended information sources (e.g., electronic databases, contact with study authors, trial registers, or other grey literature sources) with planned dates of coverage |  |  | 190-198 |
| Present draft of search strategy to be used for at least one electronic database, including planned limits, such that it could be repeated |  |  | Appendix C |
| ***STUDY RECORDS*** | | | |
| Describe the mechanism(s) that will be used to manage records and data throughout the review |  |  | 201-209 |
| State the process that will be used for selecting studies (e.g., two independent reviewers) through each phase of the review (i.e., screening, eligibility, and inclusion in meta-analysis) |  |  | 201-209 |
| Describe planned method of extracting data from reports (e.g., piloting forms, done independently, in duplicate), any processes for obtaining and confirming data from investigators |  |  | 210-224 |
| List and define all variables for which data will be sought (e.g., PICO items, funding sources), any pre-planned data assumptions and simplifications |  |  | 210-224 |
| List and define all outcomes for which data will be sought, including prioritization of main and additional outcomes, with rationale |  |  | 210-224 |
| Describe anticipated methods for assessing risk of bias of individual studies, including whether this will be done at the outcome or study level, or both; state how this information will be used in data synthesis |  |  | 227-236 |
| ***DATA*** | | | |
| Describe criteria under which study data will be quantitatively synthesized |  |  | 252-253 |
| If data are appropriate for quantitative synthesis, describe planned summary measures, methods of handling data, and methods of combining data from studies, including any planned exploration of consistency (e.g., *I* ^2^, Kendall’s tau) |  |  | 252-264 |
| Describe any proposed additional analyses (e.g., sensitivity or subgroup analyses, meta-regression) |  |  | 259-264 |
| If quantitative synthesis is not appropriate, describe the type of summary planned |  |  | 237-251 |
| Specify any planned assessment of meta-bias(es) (e.g., publication bias across studies, selective reporting within studies) |  |  | 252-264 |
| Describe how the strength of the body of evidence will be assessed (e.g., GRADE) |  |  | 266-273 |

**Appendix B****.** ICF domains including the risk factors predictive and/or associated with poor outcomes after TKA and THA

**Total joint arthroplasty**

***Participation Restriction***

- Health-related quality of life measures *(*e.g., EQ*-5D, SF-36, SF-12, etc.)*
- Etc.

***Activity Limitation***

Pre- and post-operative functional test (e.g., *WOMAC, OKS, OHS*)

- Pre and postoperative disease-or joint specific patient reported outcome measure (e.g., TUG*, SCT, 6MW, etc.)*
- Etc.

***Impairment***

- Pre- and post-operative pain measure (e.g., Likert scale, *etc*.)
- Joint mobility (*active and passive ROM*)
- Muscle strength measures
- Etc.

***Personal factors***

- Older patients (>65 y.o)
- Socioeconomic situations (e.g., *income, wealth, education, employment rate, occupation, etc*.)
- Frailty
- Level of dependency on walking aids
- Obesity
- Presence of comorbidity (such as hypertension, cardiovascular disease, osteoporosis, diabetes mellitus, etc*.)*
- Psychological vulnerability factors (e.g., *anxiety, depression, kinesiophobia, Pain catastrophizing, etc*.)
- Concomitant musculoskeletal joint complaints beyond the index joint
- Etc.

***Environmental factors***

- Family, friends, and caregiver support
- Access/availability of rehabilitation professionals, access to rehabilitation programs, wait time for surgery or rehabilitation services, access to transportation, health insurance policies/coverage, health care system/policies, health professional skills, surgeon skills.
- Etc.

**Appendix** **C.** MEDLINE full search strategy.

1 arthroplasty, replacement, hip/ or arthroplasty, replacement, knee/

2 hip prosthesis/ or knee prosthesis/

3 ((knee* or hip*) adj3 (replace* or implant* or arthroplast* or prosth*)).ti,ab,kf.

4 or/1-3

5 rehabilitation/ or "activities of daily living"/ or exp exercise therapy/ or occupational therapy/ or recreation therapy/ or rehabilitation, vocational/

6 Rehabilitation Nursing/

7 Preoperative care/ or preoperative exercise/

|  |
| --- |

8 rehabilitation.fs.

9 exp Physical Therapy Modalities/

10 motor activity/ or exercise/ or muscle stretching exercises/ or exp physical conditioning, human/ or swimming/ or exp walking/ or warm-up exercise/

11 Telerehabilitation/

12 exp Ultrasonic Therapy/

13 Cryotherapy/

14 Cryotherap*.ti,ab,kf.

15 "Recovery of Function"/

16 (rehabilitat* or exercis* or physiotherap* or therap* or hydrotherap*or pre-hab* or prehab*).ti,ab,kf.

17 (preoperat* adj3 (train* or treat* or intervent*)).ti,ab,kf.

18 (electric adj2 stimulation*).ti,ab,kf.

19 Telerehabilitation*.ti,ab,kf.

20 physical conditioning.ti,ab,kf.

21 physical activit*.ti,ab,kf.

22 stretch*.ti,ab,kf.

23 (strength adj3 (train* or treat* or intervent*)).ti,ab,kf.

24 (Resistance adj3 (train* or treat* or intervent*)).ti,ab,kf.

25 (balance adj3 (train* or treat* or intervent*)).ti,ab,kf.

26 (Continuous Passive motion or CPM).ti,ab,kf.

27 walk*.ti,ab,kf.

28 (tai ji* or tai chi* or yoga*).ti,ab,kf.

29 (recover* adj3 function*).ti,ab,kf.

30 exp Social Work/

31 exp Social Support/

32 (social adj1 (work* or support)).ti,ab,kf.

33 Patient Education as Topic/

34 exp Health Education/

35 (Patient* adj3 educat*).ti,ab,kf.

36 (health* adj3 educat*).ti,ab,kf.

37 "Physical Education and Training"/

38 orthopedic equipment/ or canes/ or crutches/ or orthotic devices/ or athletic tape/ or braces/ or walkers/

39 orthopedic equipment.ti,ab,kf.

40 cane*.ti,ab,kf.

41 crutche*.ti,ab,kf.

42 orthotic device*.ti,ab,kf.

43 athletic tap*.ti,ab,kf.

44 brace*.ti,ab,kf.

45 walker*.ti,ab,kf.

46 (psycho* adj3 (educat* or retrain* or train* or recover* or treat* or guid* or instruct* or counsel* or intervent* or manage*)).ab,ti,kf.

47 exp Psychotherapy/

48 Weight Reduction Programs/ or Weight Loss/ or obesity management/

49 ((weight or body mass or bmi) adj3 (los* or manage* or reduc* or lower)).ti,ab,kf

50 diet therapy/ or Nutrition Therapy/ or Nutritional Support/

51 ((diet or dietary or nutrit*) adj3 (counsel* or support* or modif*)).ti,ab,kf.

52 or/5-51

53 randomized controlled trial.pt.

54 controlled clinical trial.pt.

55 randomized.ab.

56 placebo.ab.

57 drug therapy.fs.

58 randomly.ab.

59 trial.ab.

60 groups.ab.

61 or/53-60

62 4 and 52 and 61

63 exp animals/ not humans.sh.

64 62 not 63

**Appendix D.** Full extraction data items

| i. Details of study | - Year-published - Journal - Trial-Registration - Study-Country - Funding - Study-Design - Study-Aim - Unit-Randomization - Sample_Size_Details - Ethics_Approval - Conflict of interest |
| --- | --- |
| ii: Study eligibility | - Inclusion criteria - Exclusion criteria |
| iii: Participants | - Number-Randomized - Number-Intervention - Number- Control /intervention2 - Received-Intervention - Received-Control / intervention 2 - Completed-Trial-Intervention - Completed-Trial-Control / intervention2. - Number missing - Reason Missing |
| iv: Participants characteristics | - Diagnosis _Duration - Poor outcome risk factor - M, R Age-Patients - Sex/Gender-Patients - Weight-patients - Height-patients - BMI-Patients - Socioeconomic-Patients - Education-patients - Ethnicity-Patients - Others - Differences between groups |
| v: Intervention | - Intervention-type / setting - Intervention-description - Intervention _timing - Intervention _follow up time point - Intervention _provider - Intervention _adherence - Control OR Intervention2_type/setting - Control OR Intervention 2_aim, description - Control OR Intervention 2_timing - Control OR Intervention 2_follow up time point - Control OR Intervention 2_provider - Control OR Intervention 2_adherence |
| vi. Outcomes | - Tool category (primary (performance-based test, functional patient reported outcome measure), secondary (health related quality of life, pain)) - Tool-description - Tool-Method - Tool validated method - Tool-Results - Tool-Blinding - Tool-Imputation of missing data - Other outcome measures |
| vii. Study conclusion | - Limitation - Conclusion |
